# Fibroblast growth factor-2-mediated protection of cardiomyocytes from the toxic effects of doxorubicin requires the mTOR/Nrf-2/HO-1 pathway

**DOI:** 10.18632/oncotarget.20558

**Published:** 2017-08-24

**Authors:** Navid Koleini, Barbara E. Nickel, Jie Wang, Zeinab Roveimiab, Robert R. Fandrich, Lorrie A. Kirshenbaum, Peter A. Cattini, Elissavet Kardami

**Affiliations:** ^1^ Institute of Cardiovascular Sciences, Albrechtsen Research Centre, Winnipeg, Manitoba, Canada; ^2^ Department of Physiology and Pathophysiology, University of Manitoba, Winnipeg, Manitoba, Canada; ^3^ Department of Human Anatomy and Cell Sciences, University of Manitoba, Winnipeg, Manitoba, Canada

**Keywords:** fibroblast growth factor 2 isoforms, doxorubicin cardiotoxicity, heme oxygenase 1 cardioprotection, Nrf-2 activation, mTOR signaling

## Abstract

**Background:**

Cardiotoxic side effects impose limits to the use of anti-tumour chemotherapeutic drugs such as doxorubicin (Dox). There is a need for cardioprotective strategies to prevent the multiple deleterious effects of Dox. Here, we examined the ability of administered fibroblast growth factor-2 (FGF-2), a cardioprotective protein that is synthesized as high and low molecular weight (Hi-, Lo-FGF-2) isoforms, to prevent Dox-induced: oxidative stress; cell death; lysosome dysregulation; and inactivation of potent endogenous protective pathways, such as the anti-oxidant/detoxification nuclear factor erythroid-2-related factor (Nrf-2), heme oxygenase-1 (HO-1) axis.

**Methods and Results:**

Brief pre-incubation of neonatal rat cardiomyocyte cultures with either Hi- or Lo-FGF-2 reduced the Dox-induced: oxidative stress; apoptotic/necrotic cell death; lysosomal dysregulation; decrease in active mammalian target of Rapamycin (mTOR). FGF-2 isoforms prevented the Dox-induced downregulation of Nrf-2, and promoted robust increases in the Nrf-2-downstream targets including the cardioprotective protein HO-1, and p62/SQSTM1, a multifunctional scaffold protein involved in autophagy. Chloroquine, an autophagic flux inhibitor, caused a further increase in p62/SQSTM1, indicating intact autophagic flux in the FGF-2-treated groups. A selective inhibitor for HO-1, Tin-Protoporphyrin, prevented the FGF-2 protection against cell death. The mTOR inhibitor Rapamycin prevented FGF-2 protection, and blocked the FGF-2 effects on Nrf-2, HO-1 and p62/SQSTM1.

**Conclusions:**

In an acute setting Hi- or Lo-FGF-2 protect cardiomyocytes against multiple Dox-induced deleterious effects, by a mechanism dependent on preservation of mTOR activity, Nrf-2 levels, and the upregulation of HO-1. Preservation/activation of endogenous anti-oxidant/detoxification defences by FGF-2 is a desirable property in the setting of Dox-cardiotoxicity.

## INTRODUCTION

Doxorubicin (Dox) is a potent chemotherapeutic drug used against many types of cancers, but is associated with numerous side effects, including an increased risk for acute and chronic cardiotoxicity leading to cardiomyopathy and heart failure [1]. Dox toxicity has been studied extensively, and multiple mechanisms are implicated including: excessive production of reactive oxygen and nitrogen species, interference with iron metabolism, mitochondrial damage, intercalation with nuclear DNA and binding to Topoisomerase II (Top-II), activation of pro-cell death pathways involving increased expression of p53 and BCL2/adenovirus E1B 19 kDa protein-interacting protein 3 (Bnip-3). In addition, dysregulation of autophagy, mitophagy and lysosomal biogenesis all contribute to Dox toxicity, causing dysfunction and loss of cardiomyocytes [1–3]. While various types of anti-oxidant therapies have been effective in preventing or attenuating Dox-cardiotoxicity in animal models, clinical trials have not provided conclusive evidence [4]. Dox-induced heart disease may be managed by using drugs such as dexrazoxane, which is believed to act by chelating iron and/or by preventing Dox-Top-II interaction, and by drugs traditionally used in the treatment for heart failure [1, 5]. There is a need for additional strategies aimed at prevention or treatment of Dox-cardiotoxicity. Endogenously expressed cardioprotective factors, such as fibroblast growth factor-2 (FGF-2), as well as endogenous cytoprotective pathways merit consideration in this context.

FGF-2 is a multifunctional protein which is expressed as high (>20 kDa, Hi-FGF-2) and low molecular weight (18 kDa, Lo-FGF-2) isoforms, products, respectively, of leucine (CUG)- or methionine (AUG)-initiated translation of the same messenger (m) RNA [6]. Hi-FGF-2 is the predominant isoform found in the human, rat and mouse heart, and like Lo-FGF-2 is detected in the intracellular as well as extracellular environment [7, 8]. Lo-FGF-2 is well documented to be cardioprotective, preventing myocardial loss and contractile dysfunction during myocardial infarction and in ischemia-reperfusion scenarios [9, 10]. Lo-FGF-2 was also shown to protect neonatal rat cardiomyocytes from Dox-induced cell death *in vitro* [11]. A non-mitogenic mutant Lo-FGF-2 has also been shown to protect isolated mouse hearts against acute Dox-induced decrease in contractility [12]. Less is known regarding the role of the Hi-FGF-2 isoform in the heart. Extracellular-acting, cell-released Hi-FGF-2 induces cardiomyocyte hypertrophy and may contribute to maladaptive chronic remodeling [7, 8]. Overexpression of Hi-, but not Lo-, FGF-2 promoted apoptosis in cardiomyocytes via an intracrine pathway [13]. There is, however, no information on the effect of exogenously administered Hi-FGF-2, compared to Lo-FGF-2, on Dox-induced cardiomyocyte damage and cell death.

Here we present evidence that Hi- or Lo- FGF-2 are equally protective against multiple aspects of acute Dox-induced toxicity in neonatal rat cardiomyocytes *in vitro*. Furthermore, the FGF-2 isoform-induced protection requires activation of endogenous cytoprotective anti-oxidant pathways such as the Nrf-2/HO-1axis.

## RESULTS

### Effect of FGF-2 isoforms on Dox-induced cardiomyocyte toxicity *in vitro*

Dox is known to induce apoptotic and necrotic death in cardiomyocytes in *in vitro* models, and *in vivo*. To recapitulate the effects of Dox *in vitro*, cardiomyocytes were exposed to 0.5 μM Dox, as used in our previous study [11].

**Figure 1:**
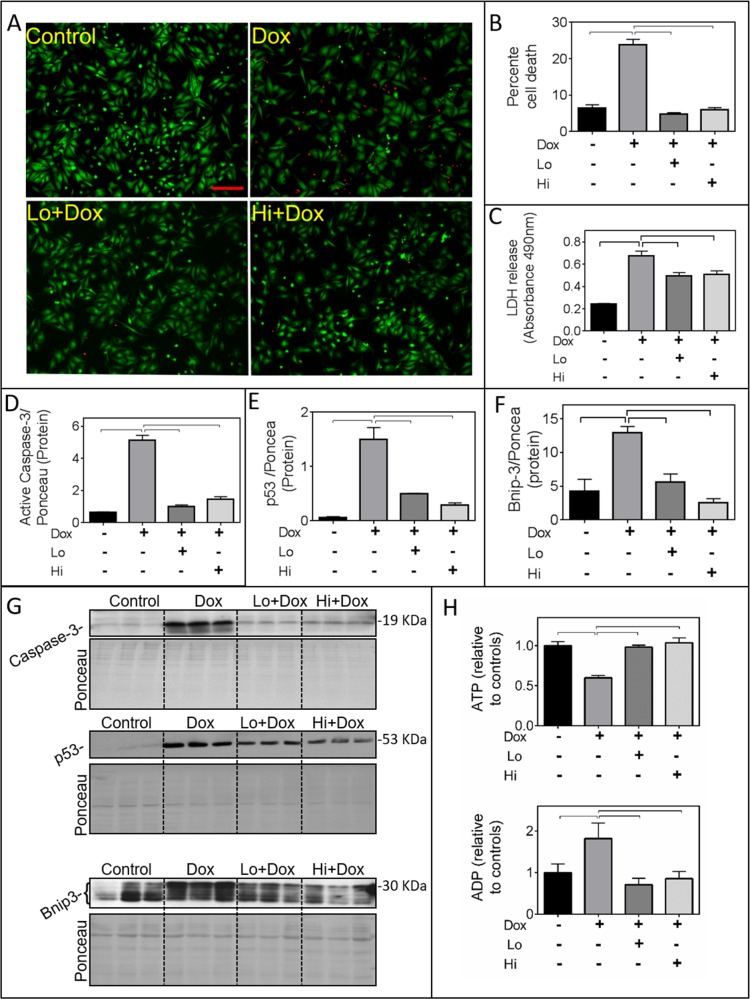
FGF-2 isoforms prevent Doxorubicin-induced toxicity in cardiomyocytes **Panels A-H** show the effects of Doxorubicin (Dox) exposure for 24 hours in the presence and absence of Lo- or Hi-FGF-2 pre-incubation, as indicated. **(A)** and **(B)** show, respectively, representative images of myocytes stained with the Live-Dead assay: Calcein-AM (green, live cells) / Ethidium homodimer (red, dead cells), and the corresponding graph with the percentage of cell death in attached cells. **(C)** LDH released in culture medium, assessed by Absorbance at 490 nm (n=6). **(D), (E), (F)** show, respectively, relative protein levels of cleaved (active) caspase-3 (19 kDa), p53 (53 kDa), and Bnip-3 (~30 kDa). Data for the graphs were obtained from the corresponding western blots shown in panel **(G)**; images of the same membranes stained for Ponceau S are also included, and served to adjust for minor variations in protein loading. **(H)** ATP and ADP levels relative to controls, as indicated (n=6). Data is plotted as mean ± SEM and statistically significant differences are shown by brackets between groups; a P<0.05 was considered significant.

Pre-incubation with recombinant Lo- or Hi-FGF-2 (10 ng/ml) for 30 minutes protected cardiomyocytes from Dox toxicity by a number of measures, assessed at 24 hours post-Dox (Figure 1). Pilot dose-response studies indicated that both FGF-2 isoforms displayed the same level of protection in the 1-100 ng/ml range, so we used the 10 ng/ml concentration for all further experiments. Based on the Live-Dead assay (Figure 1A-1B), Dox caused a significant, over 3-fold, increase in the percentage of dead cells when compared to control cultures. This effect was prevented by either Lo- or Hi-FGF-2 pre-treatment. Relative levels of LDH in the culture medium were measured, as an indicator of disruption of cardiomyocyte plasma membrane integrity. Released LDH was increased by Dox treatment, while pre-treatment with either FGF-2 isoform attenuated this increase (Figure 1C). Also, FGF-2 isoform abolished the Dox-induced upregulation in active (17 kDa) caspase-3, the tumour suppressor p53, and Bnip-3 protein levels (Figure 1D-1G), consistent with prevention of Dox-induced apoptotic and necrotic cell death. Bnip-3 immunoreactive bands migrated at 20-30 kDa, likely representing different degrees of post-translational modifications, similar to previous reports [14]; all anti-Bnip3 bands were included in our calculations. Dox caused formation of mitochondrial permeability transition pores (mPTP), as observed by the Calcein-Cobalt mPTP assay, and both FGF-2 isoforms prevented mPTP formation. Representative images are shown in Supplementary Figure 1. In addition, both FGF-2 isoforms were able to limit the Dox-induced decrease in ATP, and increase in ADP levels (Figure 1H), consistent with protective effects at the mitochondrial level. Lysates from attached cells were used for western blot-based determinations of Dox toxicities (p53, caspase 3, Bnip-3 upregulation) and the effect of both FGF-2 isoforms. We have not included detached cells which were occasionally observed in the Dox-treated samples only. As a consequence measurements of Dox toxicities by western blotting may be an underestimate of the magnitude of damage to the whole cell population; however this does not change the central observation that the FGF-2 isoforms are protective.

Dox at 0.5 μM was also found to be toxic for MCF-7 cells (a human breast cancer cell-line), as measured by Calcein-AM assay. However, unlike primary cardiomyocytes, the MCF-7 cells were not protected against Dox toxicity by either FGF-2 isoforms under the conditions tested (Supplementary Figure 2).

Overall, these observations show that both Hi- and Lo- FGF-2 can exert acute cardiomyocyte protection from Dox-induced cell death with apoptotic and necrotic features.

### Effect of FGF-2 on cardiomyocyte antioxidant/detoxification responses (Nrf-2 and downstream targets)

**Figure 2:**
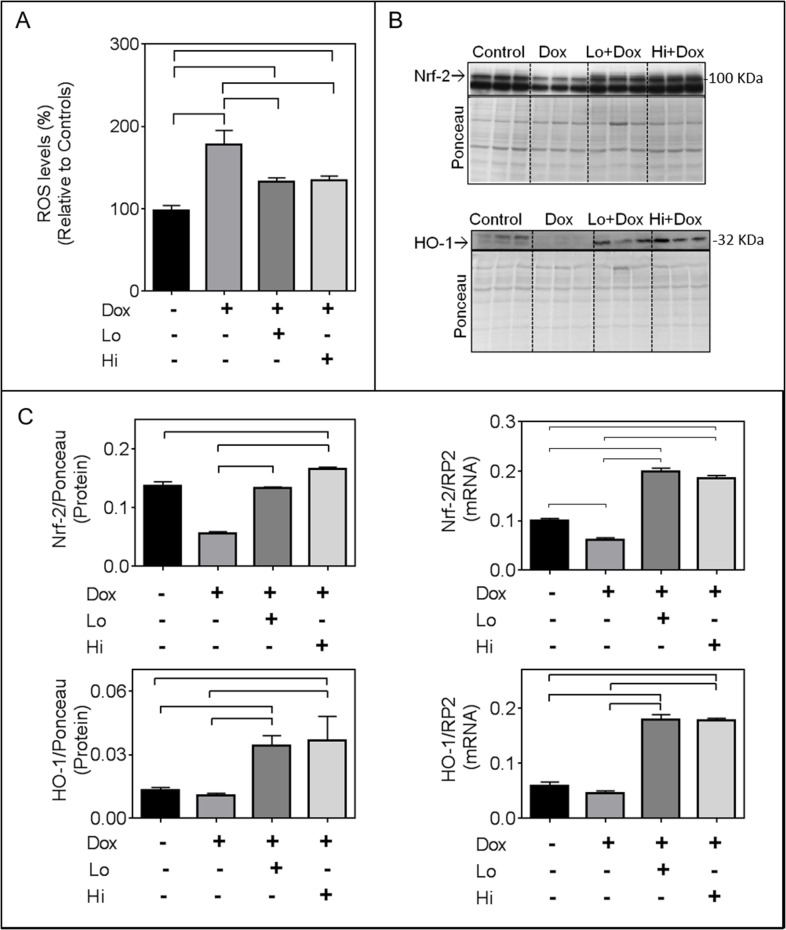
FGF-2 isoforms attenuate the effects of Doxorubicin on reactive oxygen species (ROS), Nrf-2 and its downstream target heme oxygenase 1 (HO-1) **Panel (A)** Relative ROS as measured by the fluorescence intensity of the 2′,7′ –dichlorofluorescin diacetate (DCFDA), n=8, in the absence or presence of Dox and FGF-2 isoform pre-treatment, as indicated. **(B)** Western blots for Nrf-2, and HO-1, as indicated. Nrf-2 migrates as a 100 kDa band, while HO-1 is at 32 kDa. Quantitative assessments of immunoreactive bands are included in panel C. **Panel (C)** relative Nrf-2 and HO-1 protein (n=3) as well as corresponding mRNA levels, assessed by q-PCR, (n=4), as indicated. For the mRNA or protein determinations, cardiomyocytes were exposed to Dox for, respectively, 8 or 24 hours, in the presence and absence of Lo- or Hi-FGF-2 pre-treatment. For western blot analysis, the densitometry values of the probed proteins were corrected using the densitometry values of the whole lane Ponceau S stain of the same membrane. For qPCR analysis, all target RNA levels were normalized to rat RNA polymerase II (RP2) levels. Data is plotted as mean ± SEM. Bracket indicate groups whose values are show statistically significant differences, P<0.05)

As anticipated, Dox increased levels of reactive oxygen species (ROS) in cardiomyocytes as measured by fluorescence intensity of DCF-DA (Figure 2A). This effect was attenuated by either Hi- or Lo-FGF-2 (Figure 2A). The transcription factor Nrf-2 is a master regulator of the endogenous anti-oxidant response [15]. Dox caused a reduction in the RNA and protein levels of Nrf-2; either FGF-2 isoform not only prevented this Dox-induced reduction, but also resulted in a 2-fold increase in Nrf-2 transcripts compared to controls in the presence of Dox (Figure 2C). The Dox-induced decrease in total Nrf-2 protein was prevented by either FGF-2 isoform. Relative levels of Nrf-2 protein in the presence of Hi-FGF-2 (but not Lo-FGF-2) in the Dox-treated groups were significantly higher than those of the control group; this represents the only difference between Hi- and Lo- FGF-2 activities in the present study. Please note that data shown for Nrf-2 protein represent measurements from the 100 kDa immunoreactive band, corresponding to the previously published electrophoretic migration for Nrf-2 [16]. An immunoreactive, faster migrating band was also present and may represent a truncated or modified Nrf-2. The faster band displayed the same pattern of response as the 100 kDa band, but was not included in our measurements.

Nrf-2 binds to the antioxidant response element in the promoter region of numerous target genes, including HO-1 and p62/SQSTM1. In the presence of Dox both FGF-2 isoforms significantly increased mRNA and protein levels for HO-1 relative to control cells (Figure 2B,2C). Dox alone had no significant effect on HO-1 protein levels. In the absence of Dox the FGF-2 isoforms had no effect on relative Nrf-2 and HO-1 protein levels (Supplementary Figure 3). HO-1 is a 32 kDa protein; the antibodies to HO-1detected faint immunoreactive bands at 32-34 kDa in control samples, and a 32 kDa band in the Dox/FGF-2 - exposed samples. It is possible that the 34 kDa band represents a modified HO-1. Both immunoreactive bands were included in our densitometric measurements.

**Figure 3:**
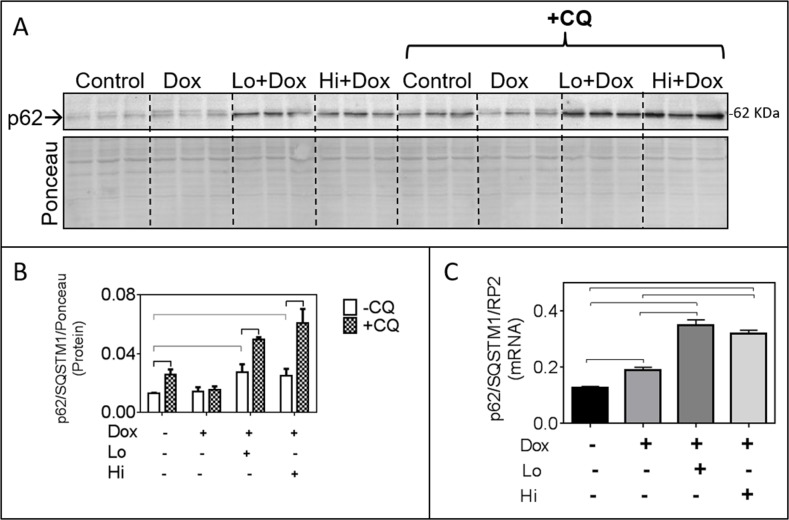
In the presence of Doxorubicin, FGF-2 isoforms promote p62/SQSTM1 upregulation which is further increased by Chloroquine (CQ) **Panel (A)** Western blot showing p62/SQSTM1 immunoreactivity, in the absence and presence of the lysosomal/autophagy flux inhibitor CQ, in cardiomyocytes exposed or not to Dox and FGF-2 pre-treatment, as indicated. Corresponding densitometric data are shown in panel B. **Panel (B)**, relative protein levels of p62/SQSTM1, in response to CQ. Densitometry of the Ponceau S (scan of the whole lane) was used to adjust for minor loading variations. **Panel (C)**, relative levels of p62/SQSTM1 mRNA in cardiomyocytes exposed, or not, to Dox and FGF-2 isoforms, as indicated; n=4. Data is plotted as mean ± SEM. For the mRNA or protein determinations, cardiomyocytes were exposed to Dox for, respectively, 8 or 24 hours, in the presence and absence of Lo- or Hi-FGF-2 pre-treatment. Rat RNA polymerase II (RP2) levels were used to normalize the target mRNA. Statistically significant differences (P<0.5) between groups are indicated by brackets.

The FGF-2 isoforms elicited significant increases in p62/SQSTM1 mRNA and protein over controls, in the presence of Dox (Figure 3A-3C). The p62/SQSTM1 is an autophagy adaptor that binds ubiquitinated items destined to be eliminated by autophagy and mitophagy [17], and represents another target of Nrf-2 [18]. Increased accumulation of p62/SQSTM1 is often interpreted as defective/blocked autophagic flux [19]. To determine if autophagic flux was blocked we examined the effect of a lysosomal/autophagy flux inhibitor, Chloroquine (CQ) on p62/SQSTM1 accumulation in the FGF-2/Dox groups. As shown in Figure 3A-3B, CQ caused additional accumulation of p62/SQSTM1 in control and FGF-2/Dox-treated groups, indicative of functional autophagic flux in these groups. CQ had no effect on p62/SQSTM1 accumulation in the presence of Dox, consistent with reports of impaired flux in this group [2, 20].

**Figure 4:**
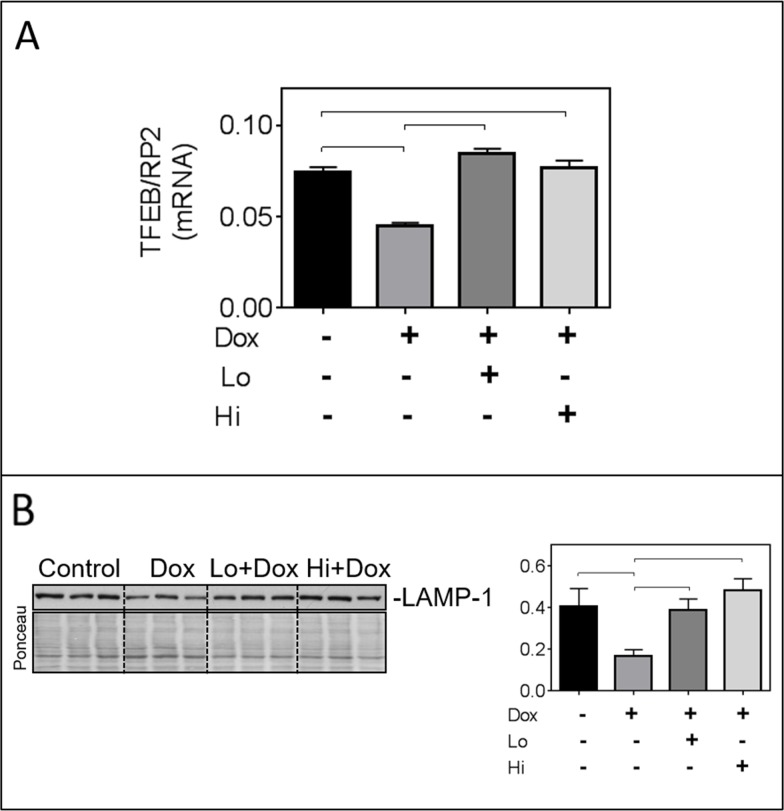
FGF-2 isoforms prevent the Dox-induced downregulation of transcription factor EB (TFEB) and lysosomal associated membrane protein-1 (LAMP-1) **Panel (A)** Relative mRNA levels of TFEB in cardiomyocytes exposed to Dox for 8 hours in the presence and absence of pre-incubation with FGF-2 isoforms. Rat RNA polymerase II (RP2) levels were used to normalize the target mRNA. **(B)** Western blot for LAMP-1, and corresponding graph after 24-hour exposure to Dox in the presence and absence of Lo- or Hi-FGF-2 pre-incubation. Ponceau S stain of the same membrane, used to correct for variations in loading. Brackets indicate groups displaying statistically significant differences.

Impaired autophagic flux can be due to defects in lysosomal biogenesis and/or function [2]. To further document an effect of Dox on lysosomes, relative levels of the mRNA for the transcription factor-EB (TFEB, a master transcription factor for lysosomal biogenesis) and of the lysosomal protein LAMP1 were assessed. Dox significantly reduced relative levels of TFEB mRNA and LAMP1 protein, and these effects were prevented by FGF-2 isoforms (Figure 4A & 4B).

### The role of mTOR and HO-1 in the FGF-2-induced protection from Dox

**Figure 5:**
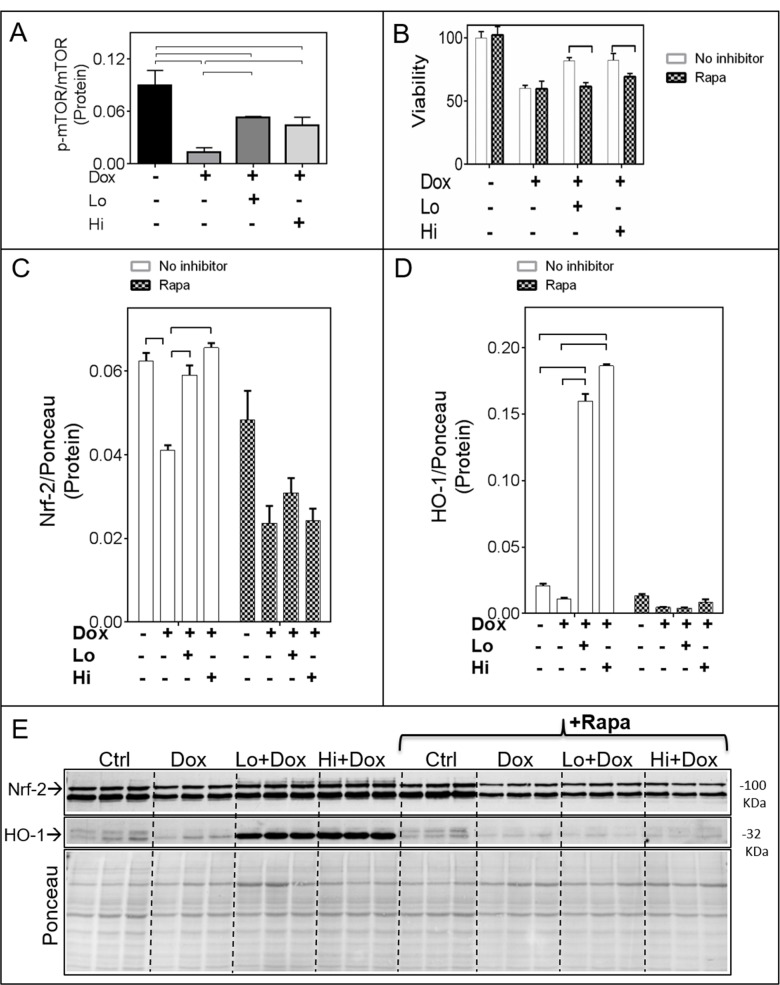
The mTOR pathway mediates the FGF-2 induced effects on Nrf-2 and HO-1 **Panel (A)** Relative levels of active (phospho-Ser2448 mTOR)/total mTOR (n=4). The corresponding western blot is shown in Supplementary Figure 4A. **(B)** Cardiomyocyte viability as estimated by the Calcein-AM (fluorescence intensity) assay, in the absence (empty columns) or presence (shaded columns) of Rapamycin, Dox, and FGF-2 isoforms (n=8), as indicated. **(C)** and **(D)**. Relative protein levels for, respectively, Nrf-2 and HO-1 in the absence (empty columns) or presence (shaded columns) of Rapamycin (n=3). Data is plotted as mean ± SEM and statistical differences are shown by brackets where significant P<0.05. The corresponding western blot is shown in **Panel (E)**.

**Figure 6:**
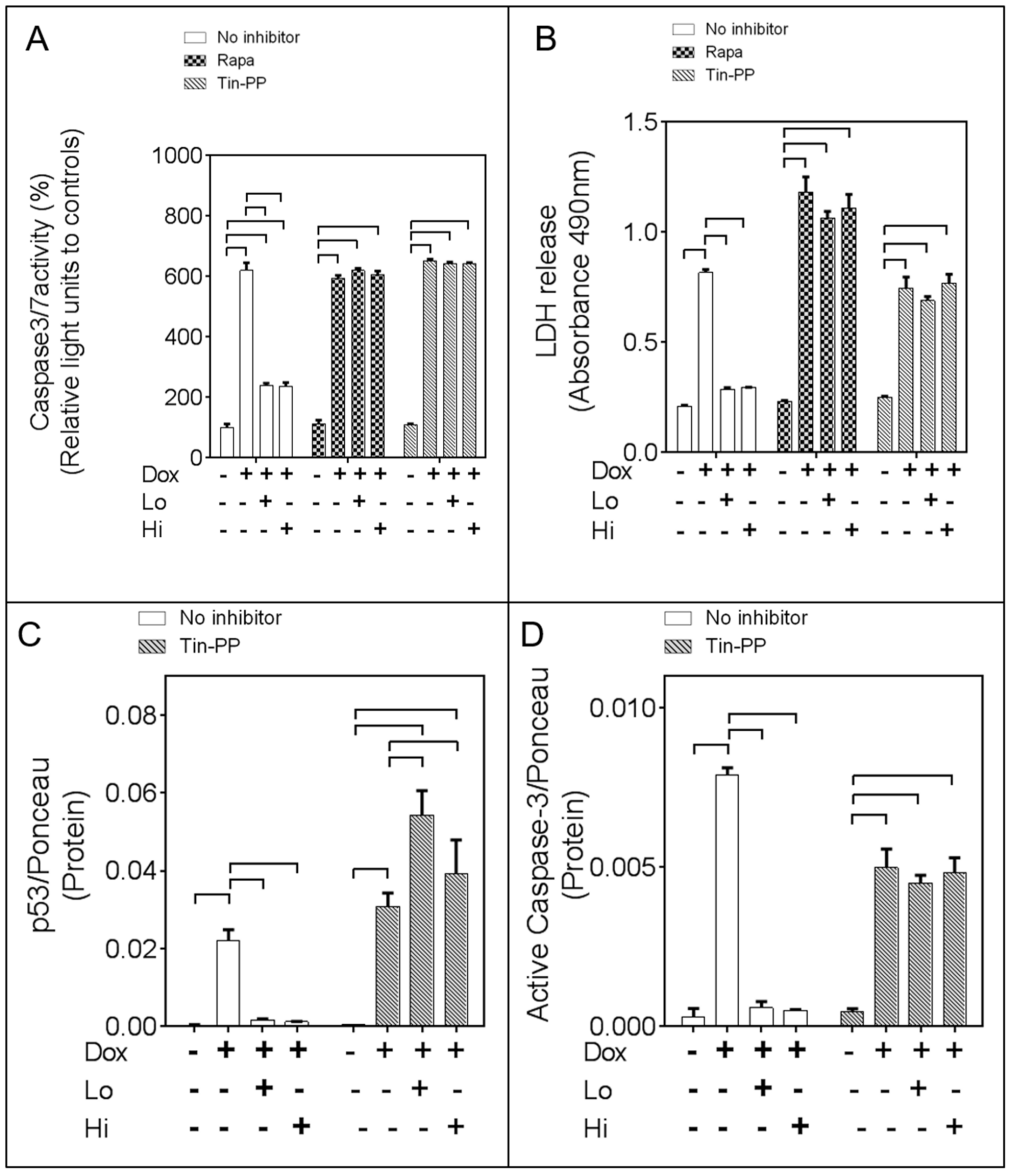
The mTOR and Heme Oxygenase-1 (HO-1) activities mediate the pro-cell survival effects of FGF-2 isoforms **Panels (A)** and **(B)**. Relative caspase 3/7 activity, and LDH release, respectively, in cardiomyocytes exposed to Dox, and FGF-2 isoform pre-treatment, in the absence or presence of Rapamycin (mTOR inhibitor) and Tin-Protorphyrin (Tin-PP), as indicated;n=4. **Panels (C)** and **(D)**. p53, or cleaved 19 kDa caspase-3 levels, respectively, in cardiomyocytes exposed to Dox, and FGF-2 isoform pre-treatment, in the absence or presence of Rapamycin and Tin-PP, as indicated, n=3. Corresponding western blots are included in Supplementary Figure 4C. Ponceau S stain of the same membrane, used to adjust for minor variations in loading. Data is plotted as mean ± SEM and brackets denote groups presenting statistically significant (P<0.05) differences.

Exposure of cardiomyocytes to Dox resulted in a significant decrease in the active (p-Ser2448)-mTORC1/total mTORC1 ratio, an effect that was limited by either Lo- or Hi-FGF-2 pre-treatments (Figure 5A and Supplementary Figure 4A). A selective inhibitor for mTORC1, Rapamycin, was used to examine whether mTOR activity mediated the protective effects of Hi- and/or Lo-FGF-2. Cells were treated with Rapamycin for 30 min prior to stimulation by FGF-2 for an additional 30 min, and then exposed to Dox for 24 hours. Rapamycin alone had no effect on cell survival in the absence or presence of Dox, but abrogated both Hi- and Lo-FGF-2 induced protection, as measured by the Calcein-AM viability assay (Figure 5B). Rapamycin prevented the FGF-2-induced restoration of relative Nrf-2 protein levels (Figure 5C and 5E). In addition, Rapamycin prevented the robust upregulation of HO-1 protein in the FGF-2/Dox groups (Figure 5D and 5E). Finally, Rapamycin prevented the protective effect of FGF-2 isoforms against Dox-induced upregulation of Caspase 3/7 activity, LDH release, and Dox-mediated upregulation of p53 as well as cleaved (active) caspase-3 (Figure 6A & 6B, and Supplementary Figure 4C). Another inhibitor of the mTOR pathway, Torin-1, was also able to prevent cardiomyocyte protection, and HO-1 upregulation by FGF-2 isoforms in the presence of Dox (Supplementary Figure 5). Thus, mTOR activity is required for protection from Dox-induced apoptotic and necrotic cell death, and HO-1 upregulation, by FGF-2.

A selective inhibitor of HO-1, Tin-Protoporphyrin (Tin-PP) was then used to determine if the protective effects of FGF-2 isoforms were mediated by HO-1. Tin-PP blocked FGF-2 protection from Dox-induced increase in caspase 3/7 activity, LDH release, p53 upregulation, and active caspase 3 (Figure 6A, 6B, and Supplementary Figure 4C).

## DISCUSSION

**Figure 7:**
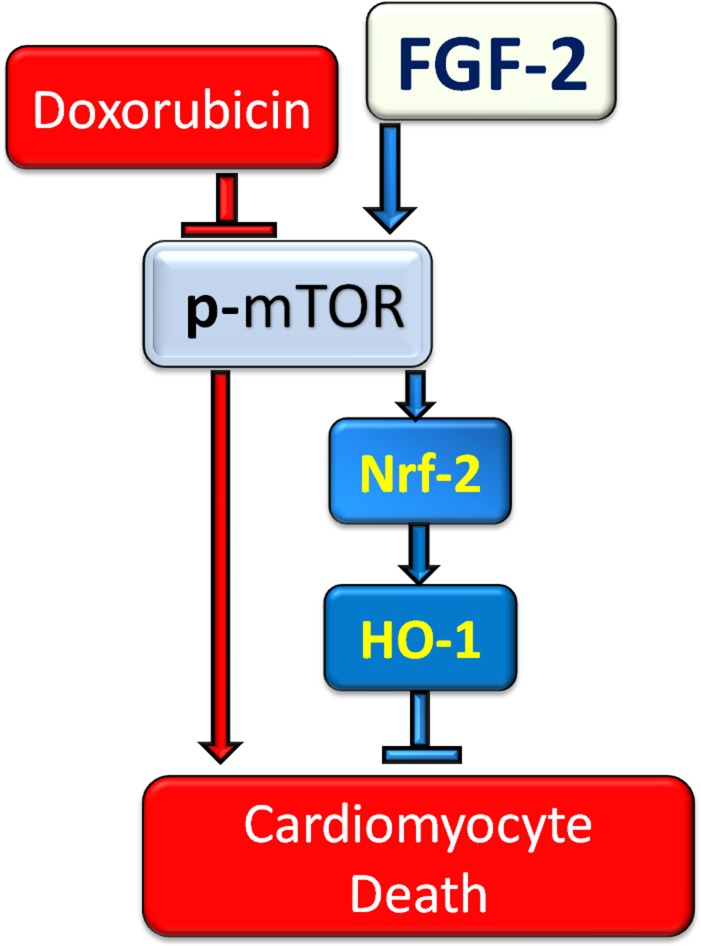
The proposed mechanism of cardioprotection against Doxorubicin by FGF-2 isoforms FGF-2 mediated protection against Dox is shown to be via restoration of Nrf-2 and robust upregulation of its target HO-1 in cardiomyocytes. mTOR re-activation is essential for FGF-2 mediated protection and restoration/upregulation of Nrf-2/HO-1.

Novel findings presented in this work are: (1) Hi-FGF-2 increases the resistance of cardiomyocytes to (acute) Dox-induced cell death and lysosomal dysregulation in a similar manner to Lo-FGF-2; (2) FGF-2 isoforms stimulate upregulation of Nrf-2 and its downstream targets HO-1 and p62/SQSTM1 in the presence, but not absence, of Dox; (3) mTOR activity is required for FGF-2 induced protection and Nrf-2, HO-1, and p62/SQSTM1 upregulation; and (4) HO-1 mediates FGF-2 protection (Figure 7).

**(i) Hi-FGF-2 protects from Dox toxicity in an acute setting**. It is well established that Dox cardiotoxicity includes mitochondrial damage, apoptotic and necrotic cell death as well as lysosomal and autophagic dysregulation [21]. In our *in vitro* model, Dox upregulated ROS, decreased cellular ATP, promoted LDH release, upregulated pro-cell death markers such as p53, Bnip-3 and active caspase 3 and caused formation of mPTP. Dox also downregulated active mTOR, which is a master regulator of growth and an inhibitor of autophagy initiation; its downregulation is expected to trigger autophagy initiation [2]. At the same time Dox caused lysosome-associated changes indicative of dysregulation, by decreasing expression of TFEB the master transcription factor for lysosomal biogenesis, and of LAMP1 protein, consistent with decreases in lysosomal numbers. Lysosomal dysregulation contributes to blocked autophagic clearance/flux in the presence of Dox resulting in proteotoxicity according to previous reports [20, 22], and confirmed in our system. Overall our model recapitulated multiple components of Dox-induced cardiotoxicity, supporting its validity in examining potential protective manipulations and associated mechanisms. It is of interest that FGF-2 isoforms did not protect a breast cancer cell line (MCF-7) from Dox toxicity, suggesting the possibility, in need of further investigation, that an FGF-2-based therapy may not affect the toxicity of anthracyclines against at least some types of cancer cells. It is not clear why MC-7 cells were not protected by FGF-2, although others have reported broadly similar findings [23]. One may speculate that although MCF-7 cells do express FGF-2 receptor 1 (FGFR1), [24] the receptor may be already fully activated by MCF-7-produced endogenous FGFs, or that it may display an aberrant pattern of activation as has been reported [25].

Both FGF-2 isoforms were found to prevent or attenuate all of the deleterious effects of Dox on cardiomyocytes. In the case of Lo-FGF-2, our results are broadly consistent with previous studies showing the protective effect of Lo-FGF in multiple scenarios of cardiomyocyte injury, as reviewed in [9], including Dox toxicity [11]. Although less information exists regarding the effects of extracellular-acting Hi-FGF-2, our previous *in vivo* studies documented that direct administration of Hi-FGF to the heart exerts short-term (one day) protection from cardiac ischemic injury and cell death. Interestingly, the protective effects of Hi-FGF-2, unlike those of Lo-FGF-2, were not sustainable at longer time points post-ischemia [26]. Another study showed that administered Hi-FGF-2 exerts acute post-conditioning-like cardioprotection against ischemia-reperfusion cardiac dysfunction and cell death [27]. Our present study reinforces the notion of acute cardiomyocyte protection by administered Hi-FGF-2, this time in the context of Dox-induced cardiotoxicity *in vitro*. It remains to be determined whether Hi- and/or Lo-FGF-2, can exert sustained protection from Dox cardiotoxicity.

**(ii) The Nrf-2/HO-1 pathway** is a major endogenous cytoprotective mechanismactivated under conditions of oxidative stress. Under non-stressed conditions, Nrf-2 is sequestered in the cytosol via its interaction with Keap-1 which also facilitates proteasomal degradation of Nrf-2. In response to oxidative stress, Nrf-2 dissociates from Keap1, translocates to the nucleus, and stimulates expression of HO-1 as well as multiple genes belonging to several anti-oxidant cell detoxification pathways [15, 28–30]. While exposure of neonatal cardiomyocytes to moderate, non-toxic, oxidative stress was reported to upregulate Nrf-2 and Nrf-2-target genes including HO-1 [31], excessive oxidative stress induced by Dox likely overwhelms the antioxidant defenses of cardiomyocytes. Indeed, we found that Dox downregulated Nrf-2 and failed to upregulate HO-1. Previous studies have shown that dysregulation of the Nrf-2/HO-1 axis contributes to Dox-induced cardiotoxicity: a lack of Nrf-2 exacerbated Dox-induced cardiotoxicity, while induction of increased expressionof Nrf-2 was protective *in vivo* [32–34]. FGF-2 isoforms, as shown here, prevented the Dox-induced effects on Nrf-2, and promoted robust upregulation of Nrf-2 targets such as HO-1 (and P62/SQSTM1), often used to demonstrate Nrf-2 activity. Our data indicate that FGF-2 protection from Dox toxicity is mediated by restoring or boosting the Nrf-2/HO-1 axis.

The competitive HO-1 inhibitor, Tin-PP, prevented the FGF-2 induced beneficial effects against cell death and damage, offering further support to this notion. The cardioprotective effect of HO-1 has been documented by multiple groups, as reviewed recently [35]. Lack of HO-1 sensitizes cardiac cells to various types of stress stimuli, including Dox and ischemia/reperfusion injury [36–38], while enhancing cardiac HO-1 expression is sufficient to blunt Dox and reperfusion damage [36, 39–43]. In view of its potent protective effects, there is strong interest in identifying drugs capable of boosting endogenous HO-1 and its downstream metabolites, such as carbon monoxide (CO) to enhance cardiac resistance to toxic conditions [35, 39, 44, 45]. In this context, FGF-2 administration to the heart may be considered as a means to upregulate HO-1 under conditions of oxidative stress.

In contrast to other cytoprotective agents like sulphoraphane [32, 33] and even FGF-1 in astrocytes [46], FGF-2 isoforms did not upregulate Nrf-2 / HO-1 protein levels under normal non-stressed conditions in cardiomyocytes, but only did so in the presence of Dox. Thus, the ability of FGF-2 pre-treatment to prevent the Dox-induced Nrf-2 loss (mRNA and protein), and to even upregulate HO-1 protein accumulation substantially above control levels likely requires additional, Dox-induced signal(s). One can speculate that since we found that FGF-2 decreased but did not eliminate ROS production by Dox, residual ROS was able to activate the Nrf-2/HO-1 anti-oxidant line of defense.

**(iii) p62/SQSTM1** is a multifunctional scaffold protein and another well known target of Nrf-2. We found that it was robustly upregulated by FGF-2 in the presence but not absence of Dox [47]. The p62/SQSTM1 accumulates at sites of autophagosome formation, and facilitates tethering of ubiquitinated cargo at the autophagosome [17]. As p62/SQSTM1 is expected to become degraded upon completion of autophagy (fusion of autophagosome with the lysosome, and degradation of cargo), its accumulation above control levels, as observed in the FGF-2/Dox groups, could be interpreted as the result of blocked autophagic flux. However, this does not appear to be the case. Firstly, we observed significant increases in p62 mRNA, consistent with Nrf-2-mediated transcription, indicative of increased de novo synthesis. Secondly, the autophagy flux inhibitor CQ elicited further, significant, increases in p62 protein accumulation, showing that autophagic flux was not blocked in the FGF-2/Dox groups. By the same criteria, autophagic flux was found to be blocked in the Dox-groups, consistent with previous reports [20]. Therefore our work indicates that FGF-2 pre-treatment corrected autophagic dysregulation, and possibly proteotoxic cell death, caused by Dox. It is possible that increased p62 levels, in an environment of functional autophagic flux, might better facilitate elimination of damaged cargo through autophagic clearance. In general agreement with our findings, it has been reported that Lo-FGF-2 protects cardiac cells against ischemia/reperfusion injury by p62/SQSTM1-mediated enhancement of ubiquitinated protein clearance [48].

**(iv) The mTOR pathway** is activated downstream of growth factor signaling [2]. mTOR is the master regulator of protein, nucleotide, and lipid synthesis and turnover and controls cell growth, differentiation, autophagy and metabolism of the cell [49]. Transgenic mice expressing a constitutively active form of mTOR are resistant to Dox cardiotoxicity, highlighting the crucial role of mTOR signaling in cardioprotection [50]. A serine/threonine protein kinase, mTOR is the active subunit of two different complexes: complex 1 (mTORC1) and complex2 (mTORC2).

The mTORC1 complex, which is inhibited by Rapamycin, promotes anabolic processes, and suppresses autophagy, while the mTORC2 complex is activated primarily downstream of PI3 kinase and is associated with cell proliferation and survival [49].

Dox-induced inhibition of mTOR in cardiomyocytes contributes to Dox toxicity [50–52]. We showed that FGF-2 attenuated the Dox-induced decrease in activated mTOR, and in turn Rapamycin (mTORC1 inhibitor), and Torin 1 (mTORC1 and mTORC2 inhibitor), prevented the FGF-2-triggered protective effects. Rapamycin abrogated the FGF-2-induced effects on Nrf-2 and its downstream targets HO-1 and p62/SQSTM1; while FGF-2 isoforms prevented the Dox-induced downregulation of active mTOR. Taken together our data indicate that the mTORC1 pathway is required for the FGF-2-induced effects on Nrf-2, HO-1, and p62/SQSTM1. A direct link may exist between mTOR and the FGF/FGFR1 axis. It is possible that mTOR may become phosphorylated/activated by direct interaction with FGFR1 and associated signals: in vascular smooth muscle cells mTOR was shown to interact directly with FGFR1 via Fibroblast Growth Factor Receptor Substrate 2 [53]. Further studies are required to address this issue.

Prolonged inhibition of mTOR by Rapamycin is reported to be protective against Dox-toxicity, by upregulating autophagy [54]. Rapamycin, as used in our system, was not protective by itself, likely because of the brevity of pre-treatment.

It is of interest that p62/SQSTM1 can also promote Nrf-2 activation by a positive feedback mechanism. It was demonstrated that mTOR phosphorylates p62/SQSTM1, thus strengthening the interaction of the latter with Keap-1. This results in dissociation and stabilization of Nrf-2, which can then translocate to the nucleus and activate expression of target genes such as HO-1 [55]. It will be important to determine whether the increased expression/accumulation of p62/SQSTM1 reported here contributed to Nrf-2 stabilization and activity.

## MATERIALS AND METHODS

This study was done according to the NIH Guide for the Care and Use of Laboratory Animals (NIH Publication, 8th Edition. Revised 2011). Approval was given by the Protocol Management and Review Committee of the University of Manitoba.

### Cultures

Hearts obtained from one day-old pups were used to isolate ventricular cardiomyocytes, which were plated at a density of 5 × 10^4^ cells/cm^2^ on collagen (Corning, #354236) coated dishes in the presence of 20% fetal bovine serum (FBS) in Hams F-10 culture medium, and allowed to attach overnight as described [57, 58]. The next day cells were incubated with low-serum medium (0.5% FBS, 1% insulin, 1% transferrin/selenium, 1% ascorbic acid, and 1% bovine serum albumin) in Dulbecco's modified Eagle's medium (DMEM) for 24 hours. Subsequently, cardiomyocyte cultures were treated, or not, with FGF-2 isoforms (10 ng/ml) for 30 minutes, followed by administration of doxorubicin (Dox; 0.5 μM). Cells were exposed to Dox for 8 or 24 hours, for extraction of RNA or protein, respectively. Inhibitors were added to the cells 30 minutes prior to FGF-2 exposure. Myocyte purity was assessed by immunofluorescence, staining myocytes for alpha-actinin which highlights the striated nature of these cells. Cultures consisted of 95 % alpha-actinin-positive cells (cardiomyocytes), and this relative composition did not change with treatments for the duration of our experiments.

### Reagents

Recombinant rat Hi- or Lo-FGF-2 were produced in-house using plasmids described in [59]. Briefly, Lo- or Hi-FGF-2 sequence was inserted into the EcoR1 site of pET19b resulting in a Histidine Tag at the N-terminal of the fusion protein. The pET vector was transformed into the expression host, Escherichia Coli (BL21(DE3)pLysS), by heat shock. The cultures were grown in the presence of 50 ug/ml carbenicilin and 34 ug/ml chloramphenicol. Overnight ExpressTM Autoinduction system (Novagen) was used according to manufacturer's instructions to induce protein expression without the need to monitor cell growth. Immobilized metal affinity chromatography (IMAC) using Nickel- sepharose (Ni-sepharose, High Performance from GE healthcare, # 17-5268-01) was used according to the manufacturer's instructions to purify proteins containing a Histidine tag. To reduce non-specific binding to beads all buffers contained 5 mM 2-mercaptoethanol and 10% glycerol. NP-40 (0.1%) was added to the binding buffer and the first wash buffer. Immidazole was removed from purified eluates by dialysis against PBS or 0.1 M NaHCO3/0.5 M NaCl. Protein concentration of the recombinant protein was calculated from the absorbance at 280 nm wavelength and the coefficient for absorbance 0.1% (rat Lo-FGF-2 Coef for Abs = 0.86, Hi-FGF-2 = 00.735). Dox was purchased from Pfizer. Chloroquine (Sigma-Aldrich, c6628) was used at a final concentration of 5 μM. Rapamycin (Cayman, CAS 53123-88-9), Tin-PP (SantaCruz, CAS 14325-05-4), and Torin1 (APExBIO, A8312) were used at concentrations of 100 nM, 10 μM, and 100 nM respectively. The following antibodies were purchased from Cell Signaling: Cleaved Caspase-3 (1:1000, #9661), p53 (1:1000, #2524), Bnip3 (1:1000, #3769), p62/SQSTM1 (1:1000, #5114), p-Ser2448-mTOR(1:1000, #2971), mTOR (1:1000, #2972). Antibodies to Nrf-2 and HO-1 were, respectively, from Proteintec(1:2000, 16396-1-AP), and Abcam: HO-1 (1:7500, ab68477). Donkey anti-rabbit (1:5,000, Jackson Immunoresearch, #711-035-152) and anti-mouse (1:5,000, Jackson Immunoresearch, #715-035-150) antibodies conjugated to horseradish peroxidase were used as secondary antibodies. Antigen-antibody complexes were detected by Pierce™ ECL Plus Western Blotting Substrate (Thermofisher, #80196).

### Calcein-AM/Ethidium homodimer viability assay

To measure cardiomyocyte viability, cells were rinsed twice with phosphate buffered saline (PBS) at 37°C, then incubated with Calcein-AM (2 μM, C3100, Thermofisher) and ethidium homodimer (2.5 μM, E1169, Thermofisher) in PBS for 30 minutes. Images were obtained with an LSM 5 PASCAL fluorescence microscope. The fluorescence intensity of Calcein-AM (485/535 nm) and ethidium homodimer (530/620 nm) were also measured using a Microplate Fluorometer (SPECTRAMAX, GEMINI XS).

### Protein (western) immunoblotting

Cells were snap-frozen using liquid nitrogen and stored at −80°C. Cells were scraped, lysed, boiled and sonicated (Vibra cell) in sodium dodecyl sulphate (SDS)/polyacrylamide gel electrophoresis (PAGE) sample buffer (1%(w/v) SDS) supplemented (1:100) with protease inhibitor cocktail (Sigma-Aldrich, #8304) and phosphatase inhibitor cocktail set II and IV (Calbiochem, #524625 and #524628). Cell homogenates were then centrifuged briefly at 21,000 g to remove cellular debris. A bicinchoninic acid assay was used to measure protein concentration in the supernatants. Following SDS-PAGE, proteins were transferred to polyvinylidene fluoride (PVDF) membranes. The PVDF membranes were stained for 5 min by 0.01% (w/v) Ponceau S (Sigma-Aldrich, P3504) in 0.15% trichloroacetic acid to assess overall protein transfer. Non-specific binding was blocked by incubation in 10% milk/TBS-T or 5% BSA/TBS-T for 1 hour at room temperature. Ponceau S (total protein estimate) was used to correct for loading variations. Densitometry values (arbitrary density units) for a particular band were divided by the corresponding densitometry values (arbitrary usints) of the Ponceau S staining of the whole lane. The Y axis in all graphs from western blot data shows relative protein levels of the groups within the graph.

### ATP/ADP assay

Luminescent ATP Detection Assay Kit (Abcam, ab113849) was used to measure the levels of ATP, ADP, and ATP/ADP ratio according to the manufacturer's protocol.

### Real-time reverse transcriptase polymerase chain reaction (qPCR)

Cardiomyocyte RNA extraction followed by qPCR was done as previously described [60]. All target RNA levels were normalized to rat RNA polymerase II (RP2) levels and shown as relative ratios. Specific primers used for amplification are listed below:

### Commercial kit-based assays

Kits were used according to the manufacturers’ instructions. Image-iT™ LIVE Mitochondrial Transition Pore Assay Kit (I35103) was used to study mitochondrial permeability transition pore opening (mPTP). DCF-DA (2′7′-dichlorodihydrofluorescein diacetate (Thermofisher, D399) was used to measure levels of ROS in cardiomyocytes. The Pierce™ LDH Cytotoxicity Assay Kit (Thermofisher, 88953) was used to measure relative levels of LDH released by the cells, as an indicator of plasma membrane damage. Caspase-Glo® 3/7 Assay kit (Promega, G8090) was used to assess activation of caspases 3 and 7, as indicators of apoptosis.

### Data analysis and statistics

Each experiment used one preparation of primary cardiomyocytes, isolated from the hearts of 36 rat pups, and distributed into several plates (either 96-well plates, or 6-well-plates of 35 mm diameter/well), that were subsequently grouped based on treatment. Group size (N=number of individual wells/plates per group) varied between 3-8, unless otherwise stated. Each experiment was repeated in its entirety at least 3 times, with independently generated cardiomyocyte cultures, again with N=3-5. Data were reproducible between experiments using different myocyte isolations. The results shown in figures represent one complete representative experimental series. GraphPad Prism 6 was used to analyze data in each experimental series. One-way or two-way ANOVA (Fisher's LSD test as post-hoc) were used as appropriate. P value <0.05 was considered significant. Data is shown as mean plus or minus standard error mean (SEM).

**Table:** 

Primer	Forward (5′→3′)	Reverse (5′→3′)
HO-1	CAGGGAAGGCTTTAAGCTGGT	GGGTTCTGCTTGTTTCGCTC
Nfe2l2 (Nrf-2)	CATTTGTAGATGACCATGAGTCGC	GCCAAACTTGCTCCATGTCC
p62/SQSTM1	AGAATGTGGGGGAGAGCGTGGC	GGGTGTCAGGCGGCTTCTCTT
RNA Polymerase-II	GGCTCTCCAGATTGCGATGTG	GCAGGTAACGGCGAATGATGATG
TFEB	CGACAACATTATGCGCCTGG	GACAGGGGTAGCGTGTTAGG

## CONCLUSION

Dox-induced cardiotoxicity remains a major concern for patients receiving chemotherapy. There is strong interest in identifying compounds and developing drugs aimed at stimulating Nrf-2 and HO-1 upregulation, as a means to elicit tissue protection from toxic stimuli including Dox [35, 56]. In this context, FGF-2 isoforms, capable of activating/boosting endogenous cardioprotective (anti-oxidant/detoxification) pathways through the mTOR-Nrf-2-HO-1 signal transduction pathway, as shown here, could be considered as naturally occurring proteins to be harnessed in strategies to elicit cardioprotection from Dox.

## SUPPLEMENTARY MATERIALS FIGURES



## Abbreviations

Dox: Doxorubicin
FGF-2: Fibroblast growth factor-2
Lo-FGF-2: Low molecular weight fibroblast growth factor-2
Hi-FGF-2: High molecular weight fibroblast growth factor-2
mTOR: mammalian target of Rapamycin
Nrf-2: nuclear factor erythroid-2-related factor
HO-1: heme oxygenase-1
ROS: reactive oxygen species
Top-II: Topoisomerase-II
Bnip-3: BCL2/adenovirus E1B 19 kDa protein-interacting protein 3
mPTP: mitochondrial permeability transition pore
TFEB: Transcription factor EB
LAMP1: Lysosomal associated membrane protein-1
Tin-PP: Tin Protoporphyrin
FGFR1: Fibroblast Growth Factor Receptor 1
FRS-2: Fibroblast Growth Factor Receptor Substrate 2
RP2: RNA polymerase II
