# Supplementary material for: Fibroblast growth factor-2-mediated protection of cardiomyocytes from the toxic effects of doxorubicin requires the mTOR/Nrf-2/HO-1 pathway
Source: Oncotarget. 2017 Aug 24;8(50):87415–30. doi: 10.18632/oncotarget.20558 (PMC5675643; doi:10.18632/oncotarget.20558)
Supplement: Supplementary file 1 [file oncotarget-08-87415-s001.pdf]

Fibroblast growth factor-2-mediated protection of cardiomyocytes from the toxic effects of doxorubicin requires the mTOR/Nrf-2/HO-1 pathway

SUPPLEMENTARY MATERIALS

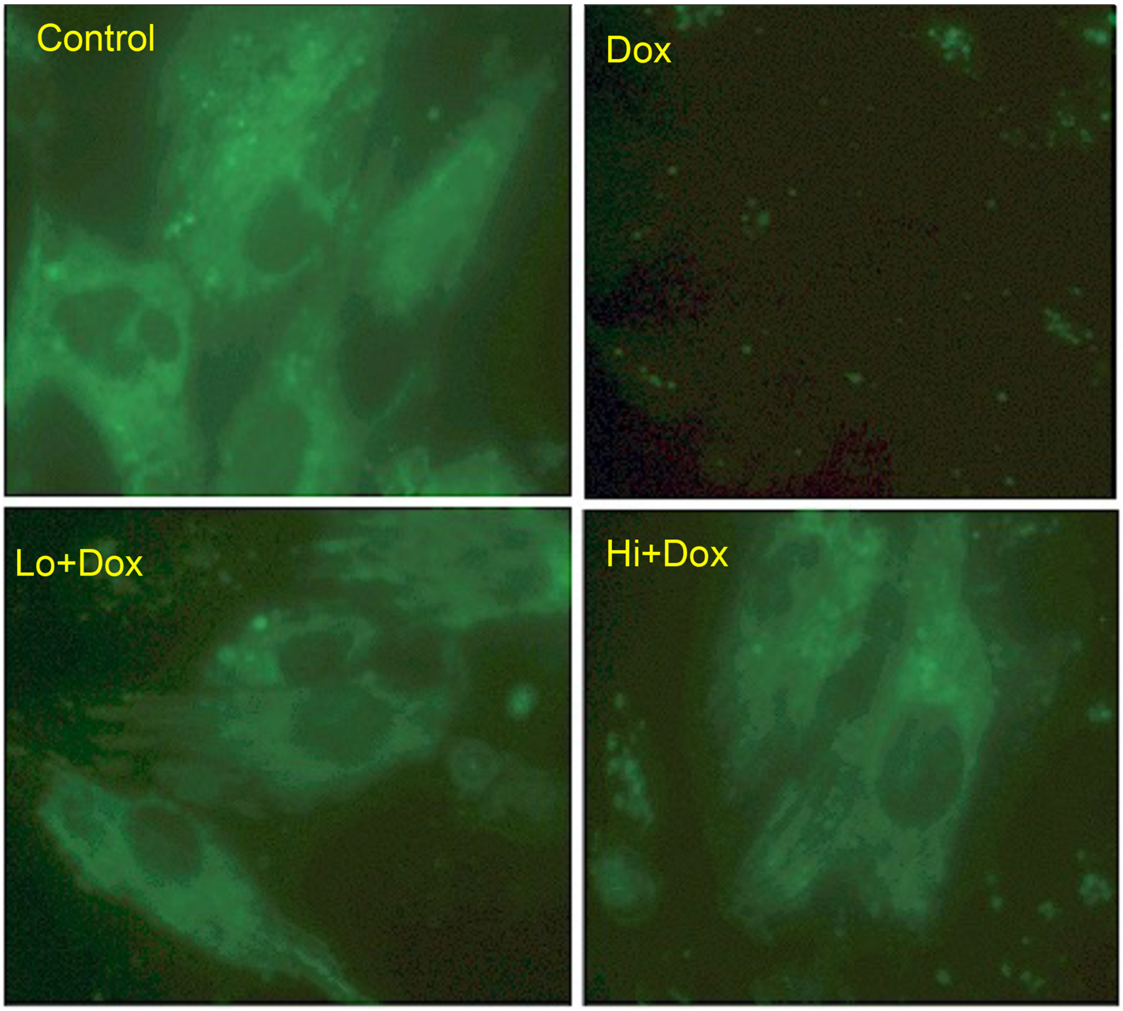

Supplementary Figure 1: FGF-2 isoforms prevent the Dox-induced mitochondrial permeability transition pore, mPTP. Representative images of cardiomyocytes, in the absence or presence of Dox and FGF-2 isoforms, as indicated, stained with Calcein-Cobalt, where healthy mitochondria display green color. Cardiomyocytes were exposed to 0.5µM of Dox for 24 hours in the presence and absence of Lo- or Hi-FGF-2 pre-incubation.

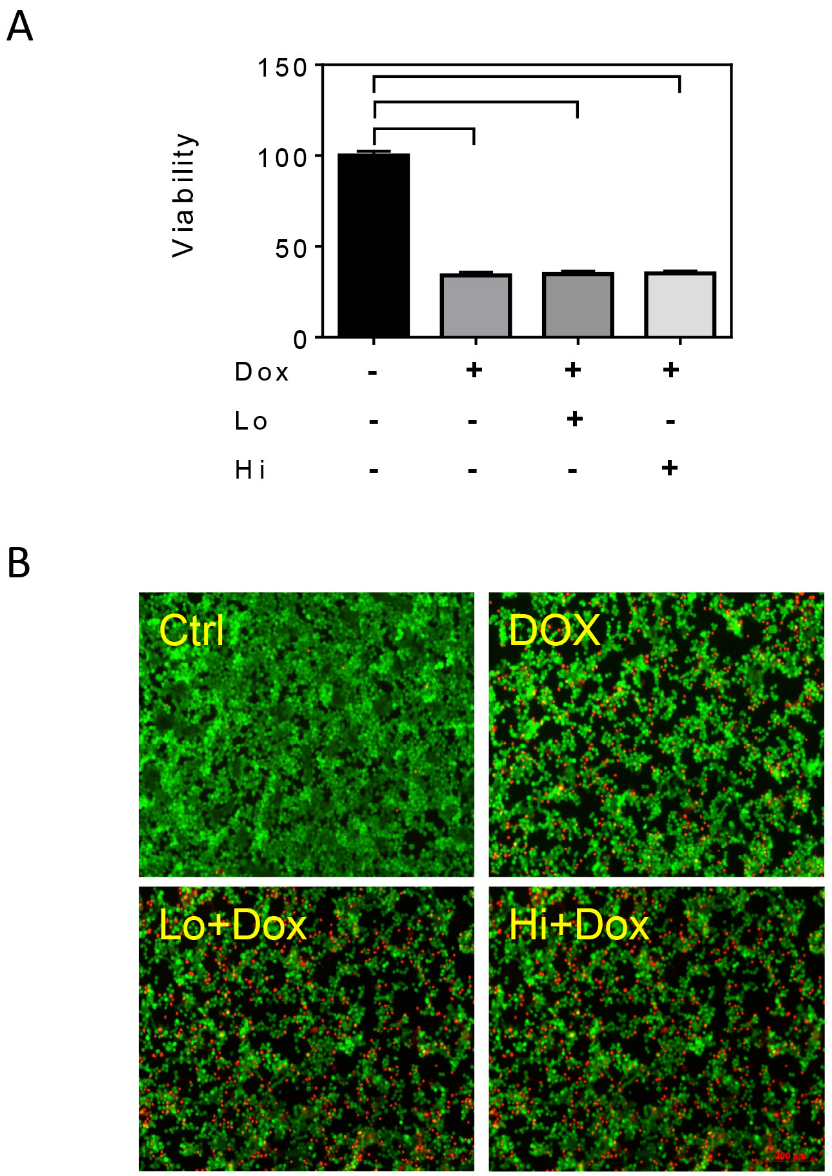

Supplementary Figure 2: FGF-2 isoforms do not protect MCF-7 cell from Dox-induced death. **(A)** Viability of MCF-7 cells, measured by the Calcein-AM fluorescence intensity assay. Cells were insulted with Dox 0.5 µM for 24 hours in the presence and absence of FGF-2 isoform pre-incubation for 30 min. **(B)**. Representative images of Calcein-AM (green, live cells)/Ethidium homodimer (red, dead cells) assay are shown in the lower section. Data is plotted as mean ± SEM and statistical differences are shown by brackets where significant $P<0.05$. The scale bar shown in the figure corresponds to 200 µm.

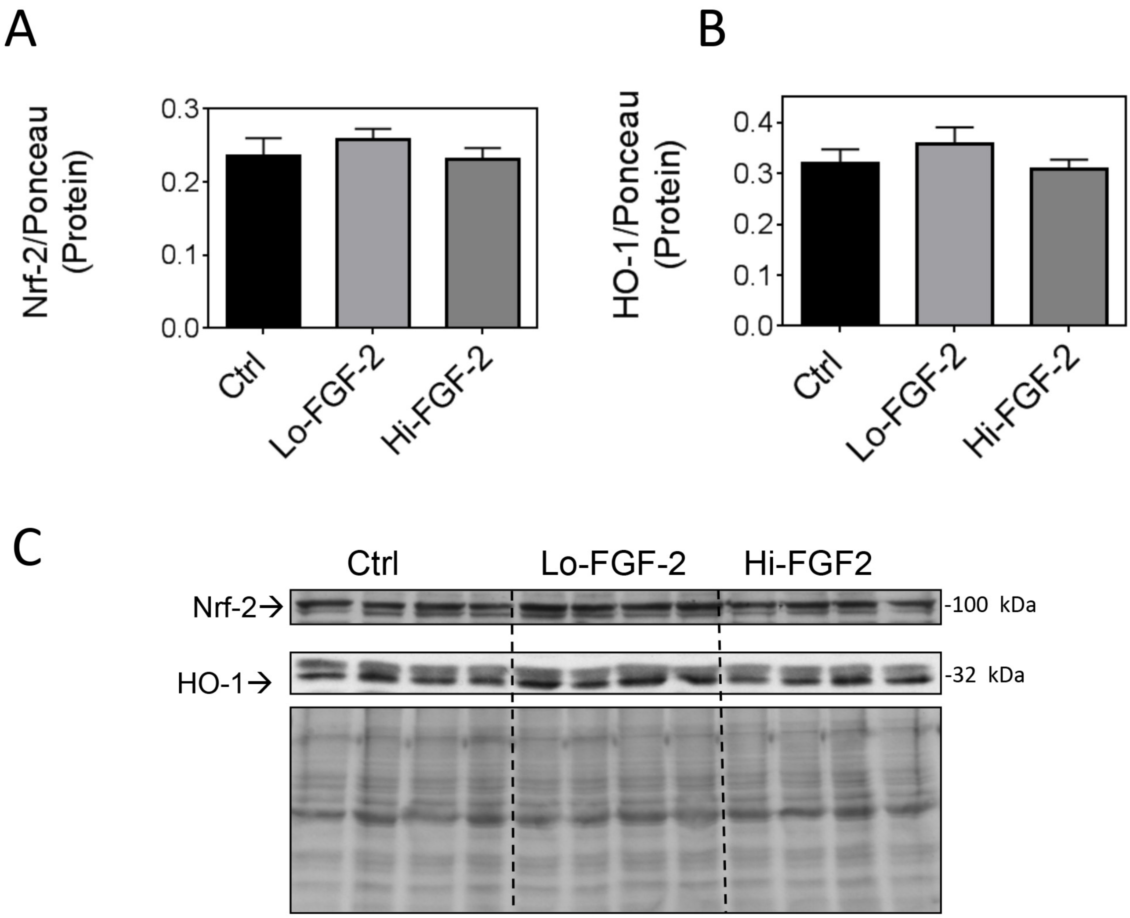

Supplementary Figure 3: FGF-2 isoforms do not change Nrf-2 and HO-1 protein levels in the absence of Doxorubicin. **(A)** and **(B)** show, respectively, relative protein levels of Nrf-2 and HO-1 in cardiomyocytes stimulated with Lo- or Hi-FGF-2 (10 ng/ml) for 24 hours. The corresponding western blots are shown in **(C)**. Ponceau S staining of the total transferred proteins was used for normalization. There are no significant differences between the groups.

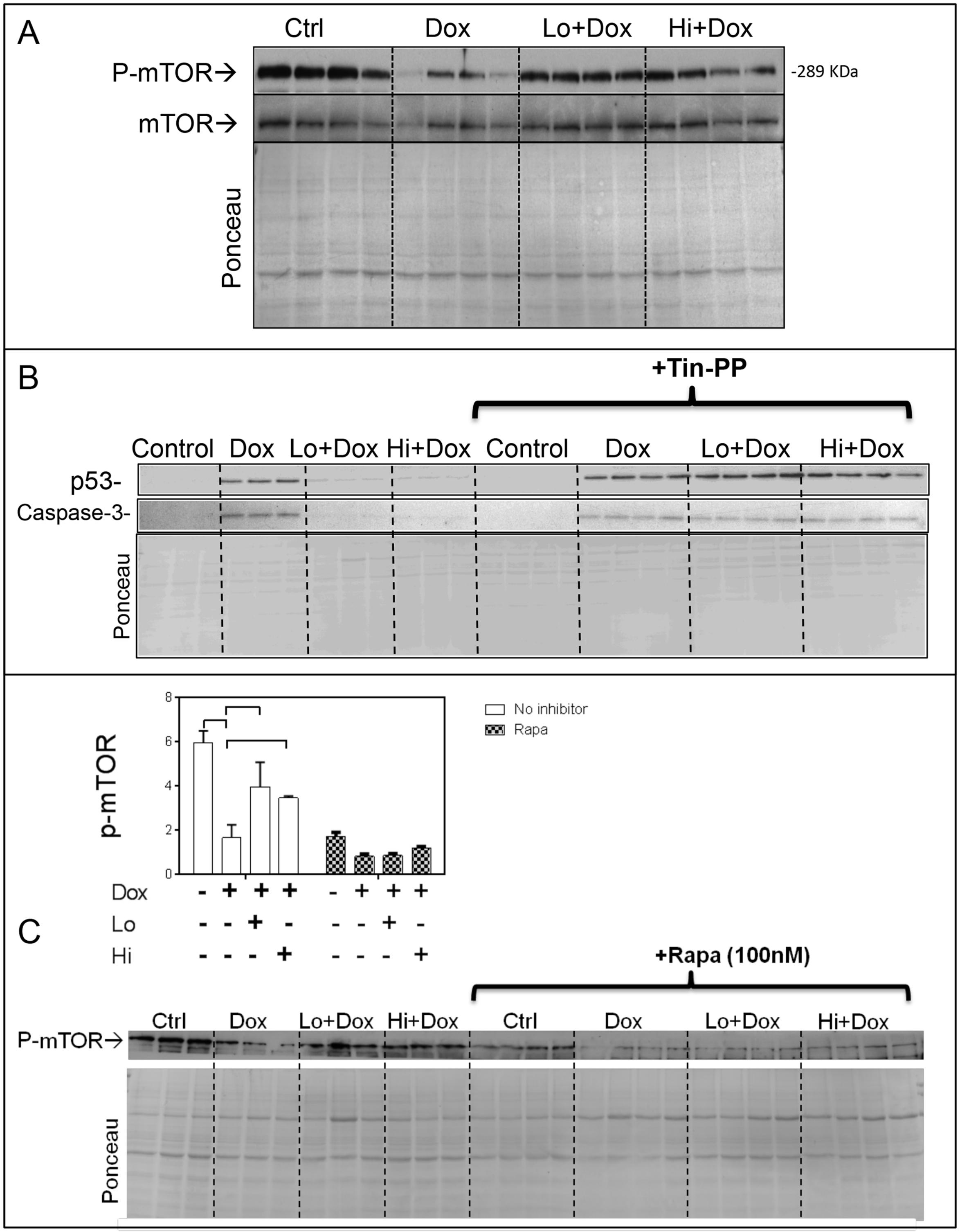

Supplementary Figure 4: Western blots of cardiomyocytes insulted with Dox for 24 hours, with and without Lo- or Hi-FGF-2 (10ng/ml) 30-min pre-incubation, in the presence and absence of Rapamycin or Tin-PP. **Panels (A)** and **(B)** show the corresponding western blots for Figures 5.A and 6.C and D, respectively. **Panel (C)** shows relative levels of p-mTOR to total loaded protein (Ponceau S) in cardiomyocytes.

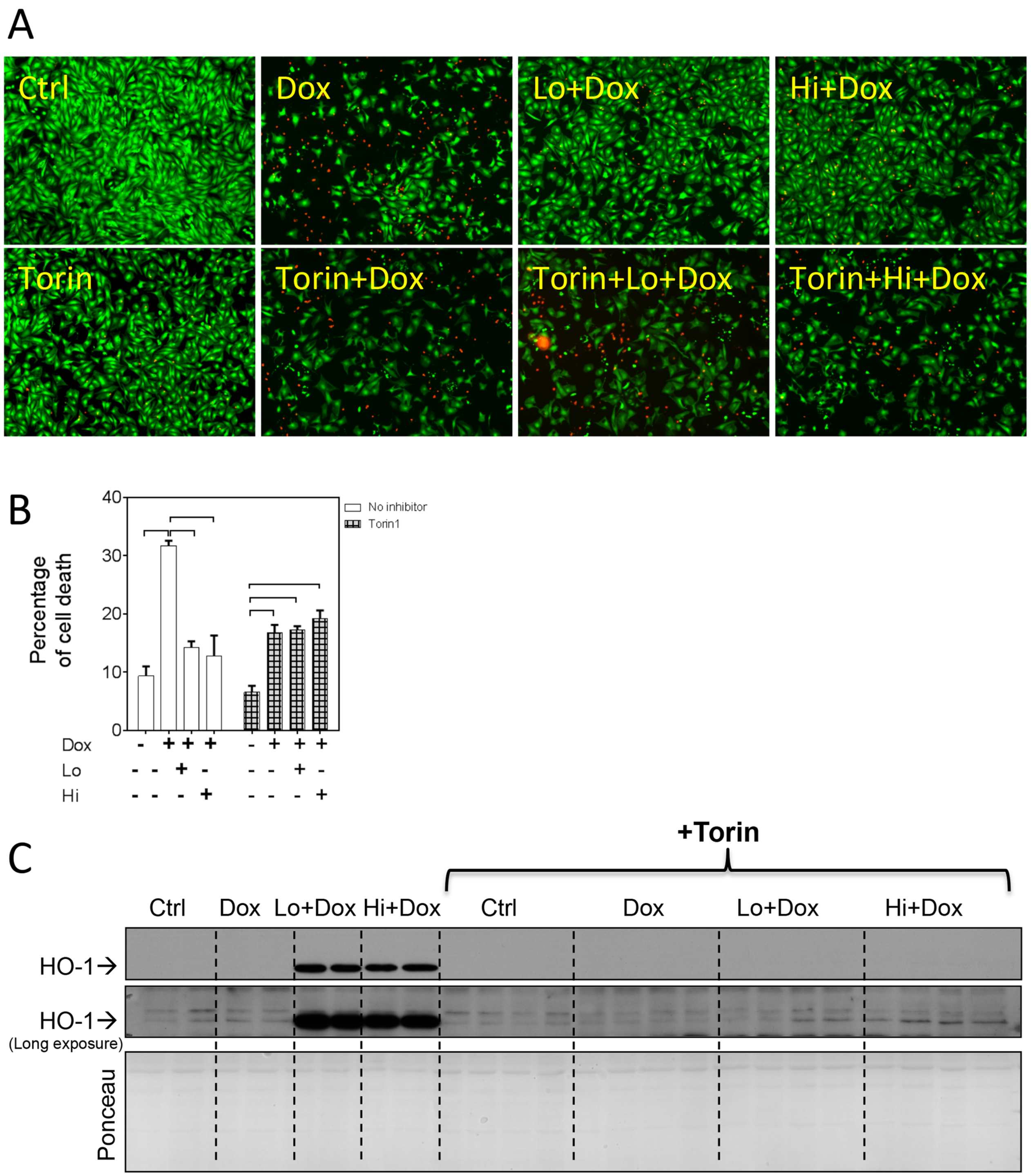

Supplementary Figure 5: Torin-1 prevents cardioprotection and upregulation of HO-1 by FGF-2 isoforms. (A) Representative fluorescence images from cardiomyocytes exposed or not to Dox, FGF-2 isoforms, and Torin-1, as indicated, stained with the Live/Dead assay. **(B)** Corresponding quantitative data (percent cell death on attached cells), n=4. **(C)** shows a western blot illustrating the effect of Torin on the FGF-2-induced upregulation of HO-1. Both short and long exposed immunoblot images are shown, and demonstrate that Torin abolished the FGF-2 isoform-induced HO-1 upregulation in the presence of Doxorubicin.
